# Correction to: Protein phosphatases 1 and 2A and their naturally occurring inhibitors: current topics in smooth muscle physiology and chemical biology

**DOI:** 10.1007/s12576-017-0577-1

**Published:** 2017-11-27

**Authors:** Akira Takai, Masumi Eto, Katsuya Hirano, Kosuke Takeya, Toshiyuki Wakimoto, Masaru Watanabe

**Affiliations:** 1 Department of Physiology, Asahikawa Medical University, Midorigaoka-Higashi 2-1-1-1, Asahikwa, 078-8510 Japan; 2 Department of Molecular Physiology and Biophysics and Sidney Kimmel Medical College, Thomas Jefferson University, Philadelphia, PA 19107 USA; 3 Department of Cardiovascular Physiology, Faculty of Medicine, Kagawa University, 1750-1 Ikenobe, Miki-cho, Kita-gun, Kagawa, 761-0793 Japan; 4 Faculty of Pharmaceutical Sciences, Hokkaido University, Kita 12, Nishi 6, Kita-ku, Sapporo, 060-0812 Japan; 5 Department of Frontier Health Sciences, Graduate School of Human Health Sciences, Tokyo Metropolitan University, Higashi-Ogu, Arakawa-ku 7-2-10, Tokyo, 116-8551 Japan

## Correction to: J Physiol Sci https://doi.org/10.1007/s12576-017-0556-6

The article Protein phosphatases 1 and 2A and their naturally occurring inhibitors: current topics in smooth muscle physiology and chemical biology, written by Akira Takai, Masumi Eto, Katsuya Hirano, Kosuke Takeya, Toshiyuki Wakimoto and Masaru Watanabe, was originally published electronically on the publisher’s internet portal (currently SpringerLink) on 5th July 2017 without open access.

With the author(s)’ decision to opt for Open Choice the copyright of the article changed on [date the updated version will be/was published] to © The Author(s) 2017 and the article is forthwith distributed under the terms of the Creative Commons Attribution 4.0 International License (http://creativecommons.org/licenses/by/4.0/), which permits use, duplication, adaptation, distribution and reproduction in any medium or format, as long as you give appropriate credit to the original author(s) and the source, provide a link to the Creative Commons license and indicate if changes were made.

The original article was corrected.

